# Induction chemotherapy combined with immunotherapy in locally advanced head and neck squamous cell carcinoma

**DOI:** 10.1186/s12885-021-08373-8

**Published:** 2021-05-27

**Authors:** Xia Li, Qigen Fang, Wei Du, Xu Zhang, Liyuan Dai, Yongming Qiao

**Affiliations:** 1 Department of Stomatology, Oral Medicine Center of The First Affiliated Hospital of Zhengzhou University, Zhengzhou, Henan People’s Republic of China; 2 Department of Head Neck and Thyroid, Affiliated Cancer Hospital of Zhengzhou University, Henan Cancer Hospital, Zhengzhou, Henan People’s Republic of China

**Keywords:** Sintilimab, TPF, Immunotherapy therapy, Head and neck squamous cell carcinoma

## Abstract

**Background:**

This study aimed to explore the efficacy and safety of sintilimab combined with induction chemotherapy (IC) in locally advanced head and neck squamous cell carcinoma (HNSCC) patients.

**Methods:**

A total of 163 patients were prospectively enrolled; 98 patients received IC only, and 65 patients received IC with sintilimab. Following neoadjuvant therapy, patients either underwent surgery (31.9%) or chemoradiotherapy (68.1%). Objective response rate (ORR), progression free survival (PFS), overall survival (OS), and toxicities between the two groups were compared.

**Results:**

The ORR in the IC group was significantly lower than that in the IC with sintilimab group (68.4% vs 84.6%, *P* = 0.019). Grade 3 or higher acute toxicity occurred in 15 (15.3%) and 12 (18.5%) patients in the IC and IC with sintilimab groups, respectively. However, this difference was not significant (*P* = 0.596). After follow-up with a median time of 28.0 months, the IC group had a 2-year PFS rate of 27% (95%CI: 18–36%), whereas the IC with sintilimab group had a 2-year PFS rate of 44% (95%CI: 32–56%), and this difference was significant (*P* = 0.041). The 2-year OS rates in the IC and IC with sintilimab groups were 61% (95%CI: 52–70%) and 70% (95%CI: 60–80%), respectively, the difference was not significant (*P* = 0.681).

**Conclusions:**

Addition of sintilimab to IC could provide longer PFS time than traditional chemotherapy regimen, without increasing the toxicity events.

## Background

Head and neck cancer is the sixth most common form of cancer, with 630,000 new cases and 350,000 deaths occuring annually worldwide [1]. Head and neck squamous cell carcinomas (HNSCC) are the most common head and neck cancer type. More than half of the patients with HNSCC present with locally advanced disease (cT1-2 N1–3 or cT3-4 N0–3) at initial diagnosis. An optimal treatment consists of multiple procedures, which includes surgery, radiotherapy, and chemotherapy [2–4]. Considering the key role of anatomical structures of the head and neck on function, induction chemotherapy (IC) is sometimes recommended in some patients based on the organ preservation concepts [5]. Although the benefit of IC remains controversial, evidence exists that overall survival (OS) can be improved if IC is administered for N2 patients [6]. Another work by Bossi et al. [7] demonstrated that if IC is used, the surgeries are less extensive and fewer patients need adjuvant postoperative radiotherapy. Additionally, the risk of distant metastasis could decrease if IC is performed [5].

Immunotherapy agents such as programmed cell death 1 (PD-1) or programmed cell death 1 ligand 1 (PD-L1) inhibitors show high efficacy and acceptable safety in HNSCC [8, 9]. Recently, the Food & Drug Administration (FDA, USA) has approved the PD-1 inhibitor pembrolizumab with or without cisplatin-based chemotherapy as the first-line therapy for recurrent or metastatic HNSCC [10]. Based on these promising results, it is worth exploring whether IC combined with an immunotherapy agent could have a better outcome and improve the prognosis.

Therefore, the goal of this study was to explore the safety and effectiveness of IC combined with sintilimab, a promising immunotherapy agent, in treating locally advanced HNSCC.

## Methods

### Ethics consideration

The study was approved by The First Affiliated Hospital of Zhengzhou University Institutional Research Committee (No.201356HN). The participants were asked to sign an informed consent form. All methods were performed in accordance with the relevant guidelines and regulations and adhered to the ethical standards of the institutional and national research committee as well as with the 1964 Helsinki Declaration (along with its later amendments or similar ethical standards).

### Patient selection

Between January 2014 and December 2020, 587 patients with untreated locally advanced HNSCC (cT1-2 N1–3 or cT3-4 N0–3 based on the 8th AJCC classification) approached our department for medical intervention. At initial assessment, 403 patients who could undergo surgery were excluded. The potential benefits and risk of immunotherapy were explained, in detail, to 184 patients who could not undergo surgical treatment. A total of 163 patients were then recruited for the present study. Among them, 98 patients agreed to have IC only and 65 patients agreed to have IC and immunotherapy (sintilimab) simultaneously. Twenty-one patients were excluded from the study as they refused any form of treatment.

Information related to demography, ECOG status, pathologic data, and follow-up of the 163 patients were collected and analyzed.

### Treatment principle

At our cancer center, systemic ultrasound, CT, MRI, and/or PET-CT examinations were routinely performed for every patient. Based on our official guideline [11], wherever possible, complete resection was usually the preferred method for all HNSCC patients. If the patient was not suitable for surgery at initial assessment due to reasons such as poor physical condition, unwillingness, or heavy tumor burden, IC plus definite chemoradiotherapy or surgery was suggested as an alternative solution. Postoperative adjuvant treatment was suggested in case of stage T3/4, cervical nodal metastasis, perineural invasion, lymphovascular invasion, extranodal extension, or positive margins. After the completion of definite treatment, patients were followed up every 3 months. Definite chemoradiotherapy referred to a combination of radiotherapy with a dose of approximately 60–66 Gy and 4–6 cycles of chemotherapy using docetaxel, platinum, and fluorouracil based on our national guideline [11].

### Induction chemotherapy and immunotherapy

The IC regimen included docetaxel (75 mg/m^2^), platinum (75 mg/m^2^), and fluorouracil (750 mg/m^2^/day for 5 days) (TPF) based on previous publications [12, 13]. Sintilimab (Innovent Biologics, Suzhou, China) was intravenously administered at a dose of 200 mg on day 1 of each cycle, once every 3 weeks.

Participants in the IC only group received two cycles of TPF, and participants in the IC with immunotherapy group received two cycles of TPF and sintilimab concurrently.

### Immunohistochemical (IHC) analysis

From July 2013, IHC analysis of p16 was performed for every HNSCC patient. The positivity of p16 overexpression was defined according to previous studies [14, 15]: ≥70% of tumor staining.

PD-L1 expression was assessed using the 22C3 pharmDx assay and calculated using the combined positive score (CPS). It was defined as the number of PD-L1-positive cells divided by the total number of tumor cells × 100; the presence of a minimum of 100 viable tumor cells in the specimen was considered to be evaluable [16].

### Study endpoints

The primary endpoint of the study was to record the objective response rate (ORR); the secondary endpoints included progression free survival (PFS), overall survival (OS), and assessing toxicities. Responses were formulated as complete response (CR), partial response (PR), stable disease (SD), and progressive disease (PD) according to the Response Evaluation Criteria in Solid Tumours (RECIST, version 1.0) [17]. These were assessed during the IC +/−immunotherapy process using imaging and endoscopy, and the overall treatment response was defined as the best response recorded from the initial treatment to the curative method. The ORR referred to the proportion of patients achieving CR or PR. Among patients receiving subsequent definite chemoradiotherapy, PFS was defined as the period from the starting date of IC to disease progression or recurrence or death from any cause. For patients receiving subsequent radical surgery, PFS was defined as the period from the starting date of IC to disease recurrence or death from any cause. OS was calculated from the starting date of IC to death from any cause. The toxicity was evaluated based on graded according to the National Cancer Institute Common Terminology Criteria for Adverse Events (version 4.0) during IC.

### Statistical analysis

The chi-square test was used to compare clinical-pathologic variables, ORR, and toxicity between the two groups; the Kaplan–Meier method was used to compare the PFS and OS between the two groups. The Cox model was subsequently used to assess the independence of immunotherapy in affecting the PFS and OS. All statistical analyses were performed using SPSS 20.0 (Chicago, USA), and *P* < 0.05 was considered to be significant.

## Results

### Baseline characteristics

Among the 163 patients selected, 113 (69.3%) were male and 50 (30.7%) were female with a mean age of 55.6 years. The primary sites affected were oral cavity in 38 (23.3%) patients, oropharynx in 56 (34.4%) patients, larynx in 37 (22.7%) patients, and hypopharynx in 32 (19.6%) patients. Clinically, the tumor stages were classified as T2 in 46 (28.2%) patients, T3 in 63 (38.7%) patients, and T4 in 54 (33.1%) patients. The cervical node stages were classified as N0 in 16 (9.8%) patients, N1 in 48 (29.4%) patients, N2 in 79 (48.5%) patients, and N3 in 20 (12.3%) patients. Thirteen (8.0%) patients had stage III disease, while 150 (92.0%) patients had stage IV disease. Sixteen (28.6%) of the 56 patients with oropharynx cancer showed positive for p16. In 65 patients with immunotherapy, 50 (76.9%) patients had a CPS ≥1 and 26 (40.0%) patients had a CPS ≥20. The two groups had similar distribution with respect to age, gender, ECOG, disease stage, primary site, and p16 expression (Table 1, all *P* > 0.05).

**Table 1:** Baseline characteristics of the included 163 patients

Variables	Total	IC* (*n* = 98)	IC + immunotherapy (*n* = 65)	*p*
Age				
< 40	15 (9.2%)	10 (10.2%)	5 (7.8%)	
≥ 40	148 (90.8%)	88 (89.8%)	60 (92.2%)	0.587
Sex				
Male	113 (69.3%)	68 (69.4%)	45 (69.2%)	
Female	50 (30.7%)	30 (30.6%)	20 (30.8%)	0.983
ECOG^#^				
0	75 (46.0%)	43 (43.9%)	32 (49.2%)	
1	88 (54.0%)	55 (56.1%)	33 (50.8%)	0.502
Smoker	100 (61.3%)	65 (66.3%)	35 (53.8%)	0.109
Drinker	73 (44.8%)	46 (46.9%)	27 (41.5%)	0.497
Primary site				
Oral cavity	38 (23.3%)	23 (23.5%)	15 (23.1%)	
Oropharynx	56 (34.4%)	35 (35.7%)	21 (32.3%)	
Larynx	37 (22.7%)	22 (22.4%)	15 (23.1%)	
Hypopharynx	32 (19.6%)	18 (18.4%)	14 (21.5%)	0.952
Tumor stage				
T2	46 (28.2%)	26 (26.5%)	20 (30.8%)	
T3	63 (38.7%)	40 (40.8%)	23 (35.4%)	
T4	54 (33.1%)	32 (32.7%)	22 (33.8%)	0.754
Neck stage				
N0	16 (9.8%)	10 (10.2%)	6 (9.2%)	
N1	48 (29.4%)	29 (29.6%)	19 (29.2%)	
N2	79 (48.5%)	43 (43.9%)	36 (55.4%)	
N3	20 (12.3%)	16 (16.3%)	4 (6.2%)	0.223
Disease stage				
III	13 (8.0%)	8 (8.2%)	5 (7.7%)	
IV	150 (92.0%)	90 (91.8%)	60 (92.3%)	0.913
p16 positivity^	16 (28.6%)	11 (31.4%)	5 (23.8%)	0.541
Reason for not surgery				
Refuse	41 (25.2%)	28 (28.6%)	13 (20.0%)	
Heavy tumor burden	88 (54.0%)	54 (55.1%)	34 (52.3%)	
Low physical status	34 (20.9%)	16 (16.3%)	18 (27.2%)	0.164

* *IC* induction chemotherapy

# *ECOG* Eastern Cooperative Oncology Group

^ Only patients with oropharynx squamous cell carcinoma were calculated

### Treatment response and definite treatment

In the IC only group, CR, PR, SD, and PD occurred in 10 (10.2%), 57 (58.2%), 25 (25.5%), and 6 (6.1%) patients, respectively. The ORR was 68.4% (Table 2). Among the 25 (25.5%) patients that underwent radical surgery, positive margin occurred in five patients. Although all patients received adjuvant radiotherapy, five patients also received adjuvant chemotherapy. Seventy-three (74.5%) patients received definite chemoradiotherapy, and residual viable tumor was observed in 30 patients. Ten patients received additional palliative chemotherapy.

**Table 2:** Objective response rate in the two groups

Responses	IC*	IC + immunotherapy	*p*
Effective^#^			
CR	10 (10.2%)	15 (23.1%)	
PR	57 (58.2%)	40 (61.5%)	
Invalid			
SD	25 (25.5%)	7 (10.8%)	
PD	6 (6.1%)	3 (4.6%)	0.019^^^

* *IC* induction chemotherapy

# *CR* complete response, *PR* partial response, *SD* stable disease, *PD* progressive disease

^ the *p* value indicated the difference of the objective response rates between the two groups

In the IC combined with immunotherapy group, CR, PR, SD, and PD occurred in 15 (23.1%), 40 (61.5%), 7 (10.8%), and 3 (4.6%) patients, respectively. The ORR was 84.6%, which was significantly higher than that in the IC only group (*P* = 0.019) (Table 2). Positive margin occurred in seven of the 27 (41.5%) patients who received radical surgery. Although all patients received adjuvant radiotherapy, seven patients also received adjuvant chemotherapy. Thirty-eight (58.5%) patients received definite chemoradiotherapy, and residual viable tumor was observed in 18 patients. The number of patients receiving additional palliative chemotherapy was five.

### Toxicity assessment

In the IC only group, the most and least common adverse events were anorexia and cough, respectively. There were 243 cases of grade 1–2 events occurring in all 98 (100%) patients and 23 cases of grade 3–4 events in 15 (15.3%) patients. In the IC with immunotherapy group, the most and least common adverse events were anorexia and dizziness, respectively. There were 185 cases of grade 1–2 events occurring in all 65 (100%) patients and 14 cases of grade 3–4 events occurring in 12 (18.5%) patients. The two groups had similar grade 3–4 event distribution (*P* = 0.596, Table 3). There were no treatment-related deaths in both groups.

**Table 3:** Adverse events in the two groups

Event	IC* (*n* = 98)		IC + immunotherapy (*n* = 65)		*p*^#^
	Grade 1–2	Grade 3–4	Grade 1–2	Grade 3–4	
Anorexia	57 (58.2%)	0	40 (61.5%)	0	
Nausea	40 (40.8%)	0	24 (36.9%)	0	
Fatigue	30 (30.6%)	0	23 (35.4%)	0	
Constipation	25 (25.5%)	0	18 (27.7%)	0	
Stomatitis	17 (17.3%)	0	17 (26.2%)	0	
Diarrhea	15 (15.3%)	0	13 (20.0%)	0	
Neutropenia	12 (12.2%)	10 (10.2%)	10 (15.4%)	4 (6.2%)	
Thrombocytopenia	11 (11.2%)	6 (6.1%)	10 (15.4%)	4 (6.2%)	
Vomiting	8 (8.2%)	0	6 (9.2%)	0	
Peripheral neuropathy	7 (7.1%)	0	6 (9.2%)	0	
Liver dysfunction	7 (7.1%)	0	6 (9.2%)	0	
Hypothyroidism	7 (7.1%)	0	6 (9.2%)	0	
Venous thrombosis	2 (2.0%)	2 (2.0%)	2 (3.1%)	2 (3.1%)	
Dizziness	2 (2.0%)	0	2 (3.1%)	0	
Cough	1 (1.0%)	0	0	0	0.596

* *IC* induction chemotherapy

#: the *p* value indicated the difference of the events of grade 3–4 between the two groups

### PFS and OS

**Fig. 1:**
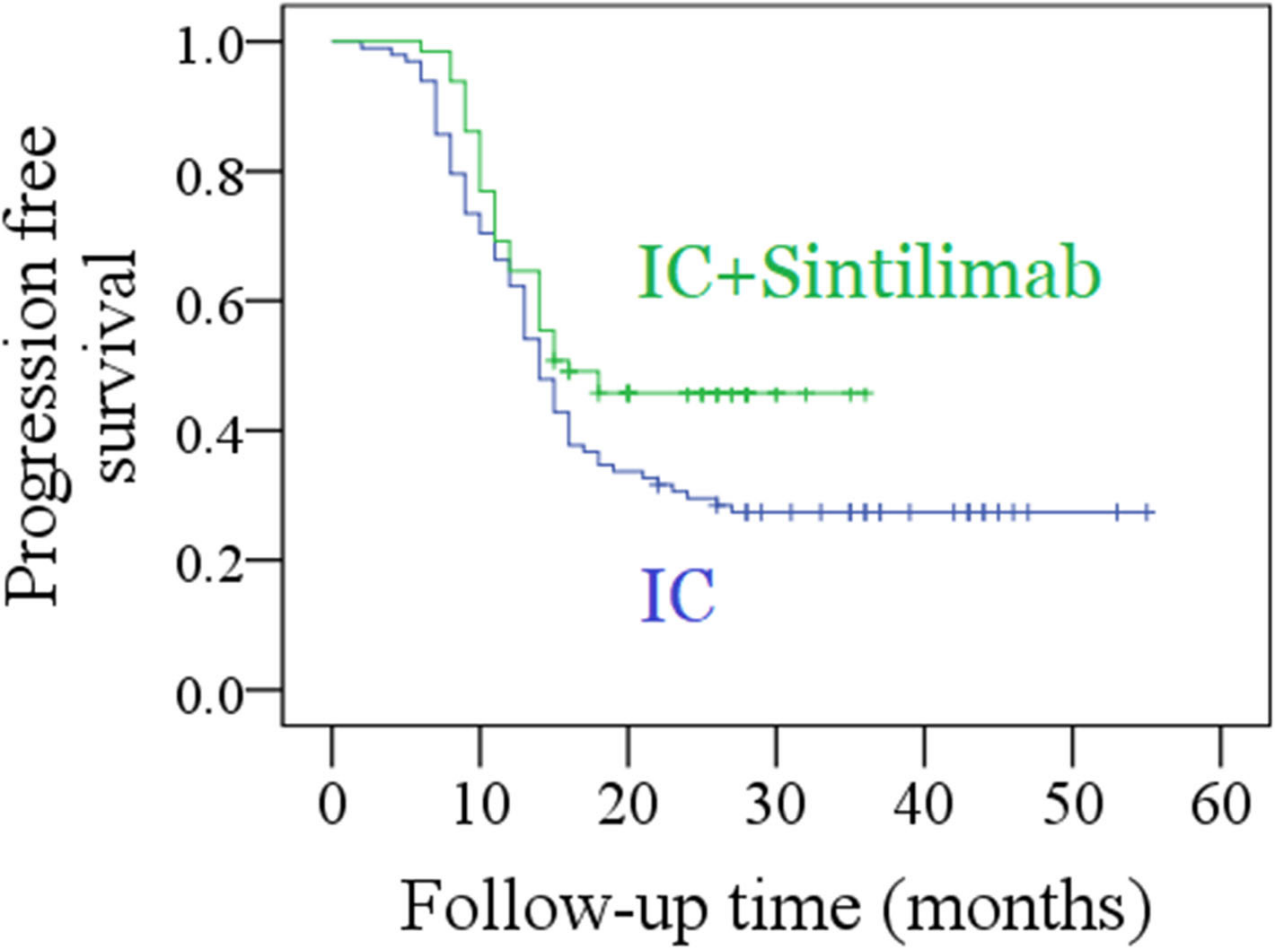
Comparison of progression free survival between induction chemotherapy (IC) group and IC combined with sintilimab group (*p* = 0.041)

In the IC only group, the median PFS time was 17.4 months, with a 2-year rate of 27% (95% CI: 18–36%). In the IC with immunotherapy group, the median PFS time was 21.1 months, with a 2-year rate of 44% (95% CI: 32–56%), which was significantly higher than that in the IC only group (*P* = 0.041, Fig. 1). Further, the Cox model confirmed the independence of immunotherapy in improving the PFS (Table 4).

**Table 4:** Univariate and multivariate analysis of prognostic factors for progression free survival

Variables	Univariate	Cox model	
	Log-rank	*p*	HR[95%CI]
Age (< 40 vs ≥40)	0.132	0.324	2.065 [0.786–4.337]
Sex (Male vs female)	0.436		
ECOG^#^ (1vs 0)	0.765		
Smoker	< 0.001	0.005	1.778 [1.221–3.998]
Drinker	0.437		
Primary site (Hypopharynx vs others)	< 0.001	< 0.001	2.876 [1.675–8.448]
Tumor stage (T4 vs T2 + T3)	< 0.001	< 0.001	2.765 [1.476–7.942]
Neck stage (N+ vs N0)	< 0.001	< 0.001	2.654 [1.664–6.831]
Disease stage (IV vs III)	< 0.001	< 0.001	3.075 [1.448–9.934]
Immunotherapy	0.041	0.014	0.781 [0.667–0.906]
Curative method (CRT vs surgery)	0.023	< 0.001	2.006 [1.478–4.643]

# *ECOG* Eastern Cooperative Oncology Group

* *CRT* Chemoradiotherapy

**Fig. 2:**
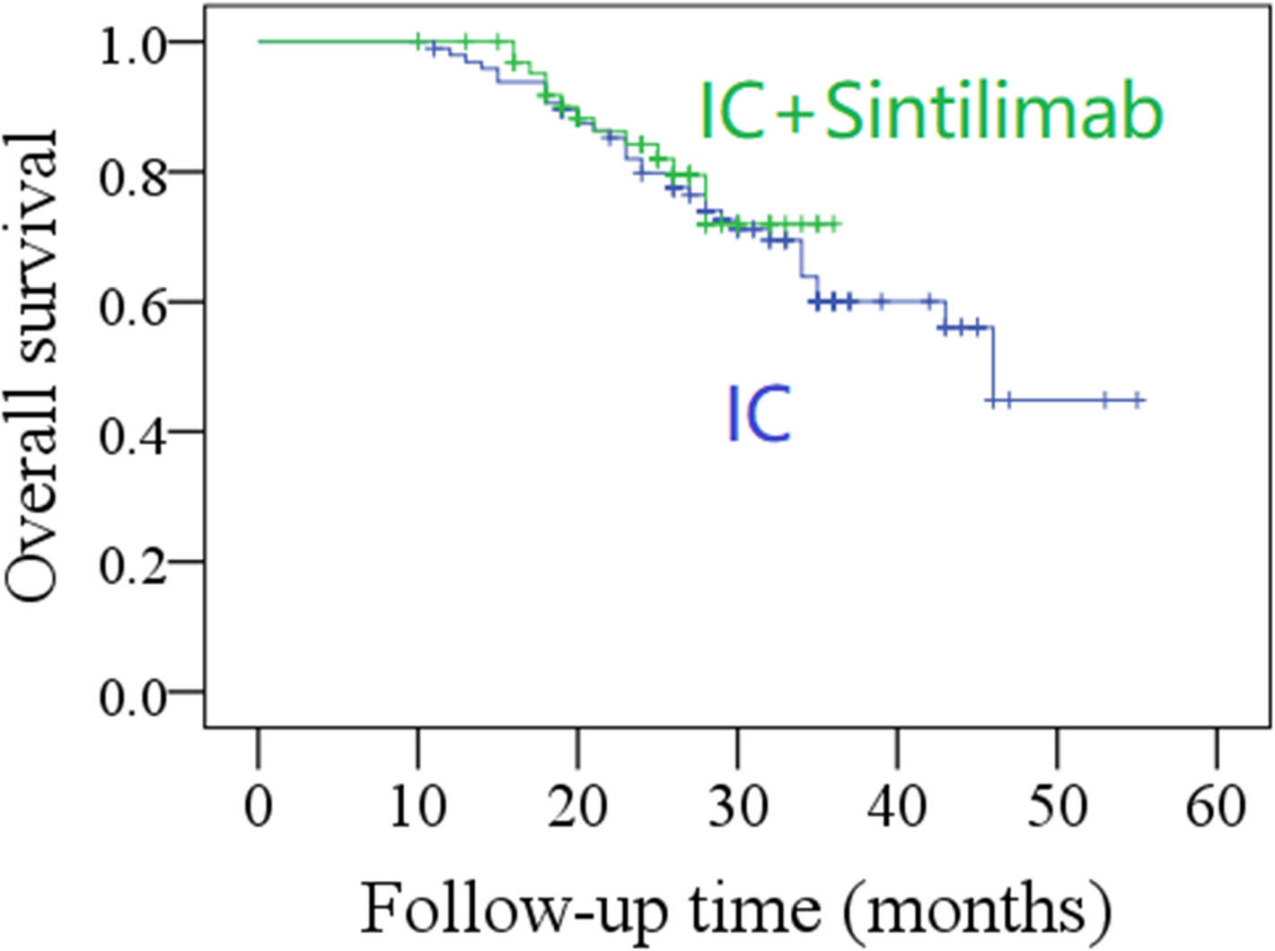
Comparison of overall survival between induction chemotherapy (IC) group and IC combined with sintilimab group (*p* = 0.681)

The median OS time in the IC only group was 36.0 months, with a 2-year rate of 61% (95% CI: 52–70%), whereas in the IC with immunotherapy group, the 2-year rate was 70% (95% CI: 60–80%). The OS time was thus similar in the two group (*P* = 0.681, Fig. 2, Table 5).

**Table 5:** Univariate and multivariate analysis of prognostic factors for overall survival

Variables	Univariate	Cox model	
	Log-rank	*p*	HR[95%CI]
Age (< 40 vs ≥40)	0.324		
Sex (Male vs female)	0.178		
ECOG^#^ (1vs 0)	0.023	0.007	1.889 [1.436–2.876]
Smoker	< 0.001	< 0.001	1.996 [1.432–3.443]
Drinker	0.435		
Primary site (Hypopharynx vs others)	< 0.001	< 0.001	3.023 [1.764–6.443]
Tumor stage (T4 vs T2 + T3)	< 0.001	< 0.001	3.453 [2.000–7.546]
Neck stage (N+ vs N0)	< 0.001	< 0.001	2.653 [1.546–6.443]
Disease stage (IV vs III)	< 0.001	< 0.001	4.532 [2.335–13.345]
Immunotherapy	0.681	0.232	2.764 [0.887–5.437]
Curative method (surgery vs CRT)	< 0.001	< 0.001	2.213 [1.233–5.432]

# *ECOG* Eastern Cooperative Oncology Group

* *CRT* Chemoradiotherapy

## Discussion

The most significant finding in the current study was that IC combined with sintilimab was associated with better ORR than IC alone, without increasing the adverse event risk in selected locally advanced HNSCC patients. The combination of IC and sintilimab provided additional survival benefits in PFS.

Major surgery is often the preferred treatment for locally advanced HNSCC [18], but many patients are not suitable for operation owing to heavy tumor burden, poor health condition, or other similar reasons at initial assessment [19]. However, IC offers another curative possibility for such patients [5, 6]. Current evidence suggests the safety and efficacy of TPF as IC in treating locally advanced HNSCC [20–22]. However, there can be further advances.

Recently, immunotherapy has attracted attention because of its promising results. Burtness et al. [8] noted that in recurrent/metastatic HNSCCs, a CPS of 20 or more in patients administered pembrolizumab alone improved OS versus cetuximab with chemotherapy (14.9 months vs. 10.7 months); a CPS of 1 or more in patients administered pembrolizumab with chemotherapy achieved a longer OS time than did the other schemes. In a similar paper by Ferris et al. [23], the two groups had comparable PFS times, but patients in the nivolumab group showed significantly longer OS time. Additionally, patients in the nivolumab group had better quality of life and physical role, and their social functioning was stable, in comparison to patients in the standard-therapy group wherein it was meaningfully worse. A later study followed a similar study design by recruiting Asian patients [24]. The authors found that nivolumab increased the OS time by nearly 3.3 months and decreased the death rate by 24.5% compared to the investigator’s choice of therapy. All these findings suggest the reliability of PD-1 inhibitors in treating recurrent or metastatic HNSCC. However, the effect of this inhibitor in primary HNSCC is largely unknown.

The potential benefits of neoadjuvant immunotherapy, including reduction of distant metastatis by early introduction of systemic therapy, have been recently reported [25]. The reduction of surgical resection extent and the intensity of adjuvant therapy by tumor downstaging and the conversion of unresectable to resectable disease are the early biomarkers for evaluation of tumor response. Carthon et al. [26] were the first to report the role of neoadjuvant immunotherapy in patients with localized urothelial carcinoma of the bladder. All 12 cases showed persistent clinical benefit for at least 3 months. Although a similar phenomenon was also observed in patients with breast cancer [27] and non-small cell lung cancer [28], literature that describes the association between neoadjuvant immunotherapy and HNSCC does not exist. Preliminary results of a few ongoing clinical trials were reported during the ASCO meeting. Ferris et al. [29] found that 11 patients (out of 23 patients enrolled initially) had nivolumab-induced preoperative tumor reduction based on computed tomography; three of them had a tumor reduction ≥40% (largest reduction being 75%). Similarly, Wise-Draper et al. [30] noted that approximately half of the initially enrolled 28 patients showed a pathologic response > 10, 32% a major response > 70%, and one had pathologic complete response after one dose of pembrolizumab. As per another report, 47% of the 21 evaluable patients had a pathologic treatment response > 10, and 40% of the patients had clinical-to-pathologic downstaging [31]. To our knowledge, the present study is the first to systemically analyze the possibility of immunotherapy as a neoadjuvant method in HNSCC patients.

As per preclinical data, sintilimab is one of the numerous PD-1 inhibitors with a binding affinity to human PD-1 that is greater than that of nivolumab and pembrolizumab, without significant pharmacokinetic behavior difference [32]. In clinical practice, sintilimab could probably prolong the PFS time by at least 2.9 months compared to traditional chemotherapy in non-small cell lung cancer [33]. In a phase 2 trial of 82 patients with classical Hodgkin lymphoma [34], 80.4% of the patients showed good treatment response. Our study confirms the efficacy of sintilimab as neoadjuvant treatment as longer PFS and higher ORR rate were observed in the TPF combined with sintilimab group. Three possible aspects based on the study by Leduc et al. [13] can probably serve as explanation for this. First, 24% of the samples showed positivity of PD-L1 (associated with better immunotherapy response) at baseline, which, after IC with TPF, increased to 71%. Second, the median density of CD8+ lymphocytes (which directly target cancer cells when activated) increased to 512 cell/mm^2^ from the initial count of 237 cells/mm^2^. Third, more patients with transformation from unresectable disease to resectable disease received radical surgery after immunotherapy. HNSCC, which has always been considered to be an immunodeficiency disease, has a natural indication for immunotherapy [10]. However, as we did not note an OS benefit with immunotherapy, some underlying mechanism needs to be explored with further in-depth studies.

Another aspect analyzed was the safety of sintilimab. In a study that enrolled 397 patients with non-small cell lung cancer, 266 patients received sintilimab combined with chemotherapy [33]. Of them, 43.2% of the patients had adverse events, with grade 3 or higher cases occurring in 5.6%; 131 patients received chemotherapy only; and 36.6% of the patients had adverse events, with grade 3 or higher cases occurring in 6.1%. The most common adverse event in the two groups was anemia and decreased neutrophil count, respectively. In another report by Shi et al. [34], wherein sintilimab was used for 96 classical Hodgkin lymphoma patients, treatment-related adverse event occurred in 93% of the cases, but most events were graded as 1 or 2. In the current study, we also noted that the two groups had similar overall adverse event incidences (grade 3 to 4), but there were no deaths. Overall, our findings suggest that sintilimab is safe to use in clinical applications.

Certain limitations of the current study must be acknowledged. First, it lacked randomization, and hence its statistical power is restricted. Second, as the follow-up time was limited, any difference regarding OS between the two groups could not be gauged as continuous regular visits were required for recording OS in patients. Finally, our sample size was relatively small. Hence, more detailed quality studies are needed to clarify the questions.

## Conclusions

In summary, the addition of sintilimab to the IC of TPF in treating patients with locally advanced HNSCC could provide a longer PFS time without elevation of adverse events. This combination regimen could provide a new treatment option for this patient population.

## Data Availability

All data generated or analyzed during this study are included in this published article. And the primary data could be achieved from the corresponding author.

## Abbreviations

IC: Induction chemotherapy
HNSCC: Head and neck squamous cell carcinoma
ORR: Objective response rate
PFS: Progression free survival
OS: Overall survival
PD-1: Programmed cell death 1
PD-L1: Programmed cell death 1 ligand 1
TPF: Docetaxel, platinum and fluorouracil

## References

[1] Ferlay J, Colombet M, Soerjomataram I, Mathers C, Parkin DM, Piñeros M, Znaor A, Bray F (2019). Estimating the global cancer incidence and mortality in 2018: GLOBOCAN sources and methods. Int J Cancer.

[2] Fang Q, Li P, Qi J, Luo R, Chen D, Zhang X (2019). Value of lingual lymph node metastasis in patients with squamous cell carcinoma of the tongue. Laryngoscope..

[3] Du W, Fang Q, Wu Y, Wu J, Zhang X (2019). Oncologic outcome of marginal mandibulectomy in squamous cell carcinoma of the lower gingiva. BMC Cancer.

[4] Cui M, Du W, Fang Q, Dai L, Qi J, Luo R (2020). Prognostic value of a family history of Oral tongue squamous cell carcinoma: a matched-pair study. Laryngoscope..

[5] Haddad RI, Posner M, Hitt R, Cohen EEW, Schulten J, Lefebvre JL, Vermorken JB (2018). Induction chemotherapy in locally advanced squamous cell carcinoma of the head and neck: role, controversy, and future directions. Ann Oncol.

[6] Marta GN, Riera R, Bossi P, Zhong LP, Licitra L, Macedo CR, de Castro JG, Carvalho AL, William WN, Kowalski LP (2015). Induction chemotherapy prior to surgery with or without postoperative radiotherapy for oral cavity cancer patients: systematic review and meta-analysis. Eur J Cancer.

[7] Bossi P, Lo Vullo S, Guzzo M, Mariani L, Granata R, Orlandi E, Locati L, Scaramellini G, Fallai C, Licitra L (2014). Preoperative chemotherapy in advanced resectable OCSCC: long-term results of a randomized phase III trial. Ann Oncol.

[8] Burtness B, Harrington KJ, Greil R, Soulières D, Tahara M, de Castro G, Psyrri A, Basté N, Neupane P, Bratland Å, Fuereder T, BGM H, Mesía R, Ngamphaiboon N, Rordorf T, Wan Ishak WZ, Hong RL, González Mendoza R, Roy A, Zhang Y, Gumuscu B, Cheng JD, Jin F, Rischin D, KEYNOTE-048 Investigators (2019). Pembrolizumab alone or with chemotherapy versus cetuximab with chemotherapy for recurrent or metastatic squamous cell carcinoma of the head and neck (KEYNOTE-048): a randomised, open-label, phase 3 study. Lancet.

[9] Cohen EEW, Soulières D, Le Tourneau C, Dinis J, Licitra L, Ahn MJ, Soria A, Machiels JP, Mach N, Mehra R, Burtness B, Zhang P, Cheng J, Swaby RF, Harrington KJ, KEYNOTE-040 investigators (2019). Pembrolizumab versus methotrexate, docetaxel, or cetuximab for recurrent or metastatic head-and-neck squamous cell carcinoma (KEYNOTE-040): a randomised, open-label, phase 3 study. Lancet..

[10] Cohen EEW, Bell RB, Bifulco CB, Burtness B, Gillison ML, Harrington KJ, Le QT, Lee NY, Leidner R, Lewis RL, Licitra L, Mehanna H, Mell LK, Raben A, Sikora AG, Uppaluri R, Whitworth F, Zandberg DP, Ferris RL (2019). The Society for Immunotherapy of Cancer consensus statement on immunotherapy for the treatment of squamous cell carcinoma of the head and neck (HNSCC). J Immunother Cancer.

[11] Guidelines Committee of Chinese society of Clinical Oncology (2020). Guideline of head and neck tumor treatment 2020.

[12] Gau M, Karabajakian A, Reverdy T, Neidhardt EM, Fayette J (2019). Induction chemotherapy in head and neck cancers: results and controversies. Oral Oncol.

[13] Leduc C, Adam J, Louvet E, Sourisseau T, Dorvault N, Bernard M, Maingot E, Faivre L, Cassin-Kuo MS, Boissier E, Dessoliers MC, Robin A, Casiraghi O, Even C, Temam S, Olaussen KA, Soria JC, Postel-Vinay S (2018). TPF induction chemotherapy increases PD-L1 expression in tumour cells and immune cells in head and neck squamous cell carcinoma. ESMO Open.

[14] Yang Y, Wang Z, Fang J, Yu Q, Han B, Cang S, Chen G, Mei X, Yang Z, Ma R, Bi M, Ren X, Zhou J, Li B, Song Y, Feng J, Li J, He Z, Zhou R, Li W, Lu Y, Wang Y, Wang L, Yang N, Zhang Y, Yu Z, Zhao Y, Xie C, Cheng Y, Zhou H, Wang S, Zhu D, Zhang W, Zhang L (2020). Efficacy and safety of Sintilimab plus Pemetrexed and platinum as first-line treatment for locally advanced or metastatic nonsquamous NSCLC: a randomized, double-blind, phase 3 study (oncology pRogram by InnovENT anti-PD-1-11). J Thorac Oncol.

[15] Wang J, Fei K, Jing H, Wu Z, Wu W, Zhou S, Ni H, Chen B, Xiong Y, Liu Y, Peng B, Yu D, Jiang H, Liu J (2019). Durable blockade of PD-1 signaling links preclinical efficacy of sintilimab to its clinical benefit. MAbs..

[16] Li P, Fang Q, Yang Y, Chen D, Du W, Liu F, Luo R (2021). Survival significance of number of positive lymph nodes in Oral squamous cell carcinoma stratified by p16. Front Oncol.

[17] Fakhry C, Lacchetti C, Rooper LM, Jordan RC, Rischin D, Sturgis EM, Bell D, Lingen MW, Harichand-Herdt S, Thibo J, Zevallos J, Perez-Ordonez B (2018). Human papillomavirus testing in head and neck carcinomas: ASCO clinical practice guideline endorsement of the College of American Pathologists Guideline. J Clin Oncol.

[18] Kulangara K, Zhang N, Corigliano E, Guerrero L, Waldroup S, Jaiswal D, Ms MJ, Shah S, Hanks D, Wang J, Lunceford J, Savage MJ, Juco J, Emancipator K (2019). Clinical utility of the combined positive score for programmed death Ligand-1 expression and the approval of Pembrolizumab for treatment of gastric Cancer. Arch Pathol Lab Med.

[19] Therasse P, Arbuck SG, Eisenhauer EA, Wanders J, Kaplan RS, Rubinstein L, Verweij J, Van Glabbeke M, van Oosterom AT, Christian MC, Gwyther SG (2000). New guidelines to evaluate the response to treatment in solid tumors. European Organization for Research and Treatment of Cancer, National Cancer Institute of the United States, National Cancer Institute of Canada. J Natl Cancer Inst.

[20] Fang QG, Shi S, Li M, Zhang X, Liu FY, Sun CF (2014). Free flap reconstruction versus non-free flap reconstruction in treating elderly patients with advanced oral cancer. J Oral Maxillofac Surg.

[21] Liu F, Yuan S, Fang Q, Sun Q (2019). Natural history of untreated squamous cell carcinoma of the head and neck. Clin Otolaryngol.

[22] Vermorken JB, Remenar E, van Herpen C, Gorlia T, Mesia R, Degardin M, Stewart JS, Jelic S, Betka J, Preiss JH, van den Weyngaert D, Awada A, Cupissol D, Kienzer HR, Rey A, Desaunois I, Bernier J, Lefebvre JL, EORTC 24971/TAX 323 Study Group (2007). Cisplatin, fluorouracil, and docetaxel in unresectable head and neck cancer. N Engl J Med.

[23] Posner MR, Hershock DM, Blajman CR, Mickiewicz E, Winquist E, Gorbounova V, Tjulandin S, Shin DM, Cullen K, Ervin TJ, Murphy BA, Raez LE, Cohen RB, Spaulding M, Tishler RB, Roth B, Viroglio Rdel C, Venkatesan V, Romanov I, Agarwala S, Harter KW, Dugan M, Cmelak A, Markoe AM, Read PW, Steinbrenner L, Colevas AD, Norris CM, Haddad RI, TAX 324 Study Group (2007). Cisplatin and fluorouracil alone or with docetaxel in head and neck cancer. N Engl J Med.

[24] Lorch JH, Goloubeva O, Haddad RI, Cullen K, Sarlis N, Tishler R, Tan M, Fasciano J, Sammartino DE, Posner MR, TAX 324 Study Group (2011). Induction chemotherapy with cisplatin and fluorouracil alone or in combination with docetaxel in locally advanced squamous-cell cancer of the head and neck: long-term results of the TAX 324 randomised phase 3 trial. Lancet Oncol.

[25] Ferris RL, Blumenschein G, Fayette J, Guigay J, Colevas AD, Licitra L, Harrington K, Kasper S, Vokes EE, Even C, Worden F, Saba NF, Iglesias Docampo LC, Haddad R, Rordorf T, Kiyota N, Tahara M, Monga M, Lynch M, Geese WJ, Kopit J, Shaw JW, Gillison ML (2016). Nivolumab for recurrent squamous-cell carcinoma of the head and neck. N Engl J Med.

[26] Kiyota N, Hasegawa Y, Takahashi S, Yokota T, Yen CJ, Iwae S, Shimizu Y, Hong RL, Goto M, Kang JH, Sum Kenneth Li W, Ferris RL, Gillison M, Namba Y, Monga M, Lynch M, Tahara M (2017). A randomized, open-label, phase III clinical trial of nivolumab vs. therapy of investigator's choice in recurrent squamous cell carcinoma of the head and neck: a subanalysis of Asian patients versus the global population in checkmate 141. Oral Oncol.

[27] Hanna GJ, Adkins DR, Zolkind P, Uppaluri R (2017). Rationale for neoadjuvant immunotherapy in head and neck squamous cell carcinoma. Oral Oncol.

[28] Carthon BC, Wolchok JD, Yuan J, Kamat A, Ng Tang DS, Sun J, Ku G, Troncoso P, Logothetis CJ, Allison JP, Sharma P (2010). Preoperative CTLA-4 blockade: tolerability and immune monitoring in the setting of a presurgical clinical trial. Clin Cancer Res.

[29] McArthur HL, Diab A, Page DB, Yuan J, Solomon SB, Sacchini V, Comstock C, Durack JC, Maybody M, Sung J, Ginsberg A, Wong P, Barlas A, Dong Z, Zhao C, Blum B, Patil S, Neville D, Comen EA, Morris EA, Kotin A, Brogi E, Wen YH, Morrow M, Lacouture ME, Sharma P, Allison JP, Hudis CA, Wolchok JD, Norton L (2016). A pilot study of preoperative single-dose Ipilimumab and/or Cryoablation in women with early-stage breast Cancer with comprehensive immune profiling. Clin Cancer Res.

[30] Forde PM, Chaft JE, Smith KN, Anagnostou V, Cottrell TR, Hellmann MD, Zahurak M, Yang SC, Jones DR, Broderick S, Battafarano RJ, Velez MJ, Rekhtman N, Olah Z, Naidoo J, Marrone KA, Verde F, Guo H, Zhang J, Caushi JX, Chan HY, Sidhom JW, Scharpf RB, White J, Gabrielson E, Wang H, Rosner GL, Rusch V, Wolchok JD, Merghoub T, Taube JM, Velculescu VE, Topalian SL, Brahmer JR, Pardoll DM (2018). Neoadjuvant PD-1 blockade in Resectable lung Cancer. N Engl J Med.

[31] Ferris RL, Gonçalves A, Baxi SS, Martens UM, Gauthier H, Langenberg M (2017). An open-label, multicohort, phase 1/2 study in patients with virusassociated cancers. (CheckMate 358): Safety and efficacy of neoadjuvant nivolumab in squamous cell carcinoma of the head and neck. (SCCHN). Ann Oncol.

[32] Wise-Draper TM, Old MO, Worden FP, O’Brien PE, Cohen EEW, Dunlap N (2018). Phase II multi-site investigation of neoadjuvant pembrolizumab and adjuvant concurrent radiation and pembrolizumab with or without cisplatin in resected head and neck squamous cell carcinoma. J Clin Oncol.

[33] Uppaluri R, Zolkind P, Lin T, Nussenbaum B, Jackson RS, Rich J (2017). Neoadjuvant pembrolizumab in surgically resectable, locally advanced HPV negative head and neck squamous cell carcinoma (HNSCC). J Clin Oncol.

[34] Shi Y, Su H, Song Y, Jiang W, Sun X, Qian W, Zhang W, Gao Y, Jin Z, Zhou J, Jin C, Zou L, Qiu L, Li W, Yang J, Hou M, Zeng S, Zhang Q, Hu J, Zhou H, Xiong Y, Liu P (2019). Safety and activity of sintilimab in patients with relapsed or refractory classical Hodgkin lymphoma (ORIENT-1): a multicentre, single-arm, phase 2 trial. Lancet Haematol.

